# Reviewing findings on the polypeptide sequence of the SARS-CoV-2 S-protein to discuss the origins of the virus

**DOI:** 10.2217/fvl-2021-0233

**Published:** 2022-04-05

**Authors:** Alberto Maria Cattaneo

**Affiliations:** ^1^Department of Plant Protection Biology, Swedish University of Agricultural Sciences, Chemical Ecology Group, Lomma, Box 190 234 22, Sweden; ^2^University of Lausanne, Center for Integrative Genomics, Lausanne, CH-1015, Switzerland

**Keywords:** coronavirus, polypeptide sequence alignment, RaTG13, receptor-binding motif, S-protein, SARS-CoV-2

## Abstract

Several investigations suggested origins of SARS-CoV-2 from the recombination of coronaviruses of various animals, including the bat *Rhinolophus affinis* and the pangolin *Manis javanica*, despite the processes describing the adaptation from a reservoir of animals to human are still debated. In this perspective, I will remark two main inconsistencies on the origins of SARS-CoV-2: polypeptide sequence alignment of the S-proteins does not return the expected identity of the receptor-binding motif among most of pangolin-CoVs and SARS-CoV-2; accurate referencing for samplings and sequencing deposition of the ancestral bat coronavirus named RaTG13 was missing since the first reports on the SARS-CoV-2 coronavirus. This contribution aims to stimulate discussion about the origins of SARS-CoV-2 and considers other intermediate hosts as a reservoir for coronavirus.

The emerging pathogenic coronavirus reported on December 2019 in China has now affected 435,626,514 people and caused over 5,952,215 deaths all around the world (last WHO COVID-19 dashboard, 1 March 2022, 05:37 pm CET – https://covid19.who.int/).

Among the first cases, some were reported from a local seafood market of Wuhan city (Hubei province, China) when six seafood street sellers with severe pneumonia were admitted to the intensive care unit of the Wuhan Jin-Yin-Tan Hospital. Metagenomics analysis of their samples (WIV04, EPI_ISL_402124 and WIV02, WIV05, WIV06, WIV07: GISAID accession numbers EPI_ISL_402127–402130) demonstrated 99.9% identity among each other and matching with 79.6% identity with the SARS coronavirus CoV-BJ01 (GenBank accession number AY278488.2) [1]. Phylogenetic comparison with the nucleotide sequences of several complete genomes of coronaviruses identified bat coronaviruses among the closest related with this new SARS-pathogen. Among these viruses, the Bat-CoV RaTG13 (GISAID EPI_ISL_402131), detected by the same research group on 2013 in fecal swabs of *Rhinolophus affinis* bats from the Chinese province of Yunnan (note: no reference was reported in Zhou *et al.* [1]), represented the most identical coronavirus to the sequenced samples (96.2% identity). The Coronaviridae Study Group of the International Committee on Taxonomy of Viruses recognized this new pathogen as a new coronavirus forming a sister clade to the prototype human and bat SARS coronaviruses (SARS-CoVs) and designates it as SARS-CoV-2 [2].

The most reliable investigations on the SARS-CoV-2 origins suggest adaptation of this human coronavirus from an animal reservoir, which supposedly underwent some sort of recombination. However, the processes describing how the adaptation of the virus from an animal to a human host may have occurred still cannot be explained, becoming a matter of a wide discussion in both the society and in the scientific community.

By reviewing the ongoing knowledge on SARS-CoV-2 origins, and analyzing a polypeptide sequence alignment of the S-proteins of several SARS-coronaviruses, this contribution aims to stimulate further questions about the origins of both this virus and its most closely related RaTG13.

## Investigating the origins of the amino acid insertions within the SARS-CoV-2 S-protein

**Figure 1.**
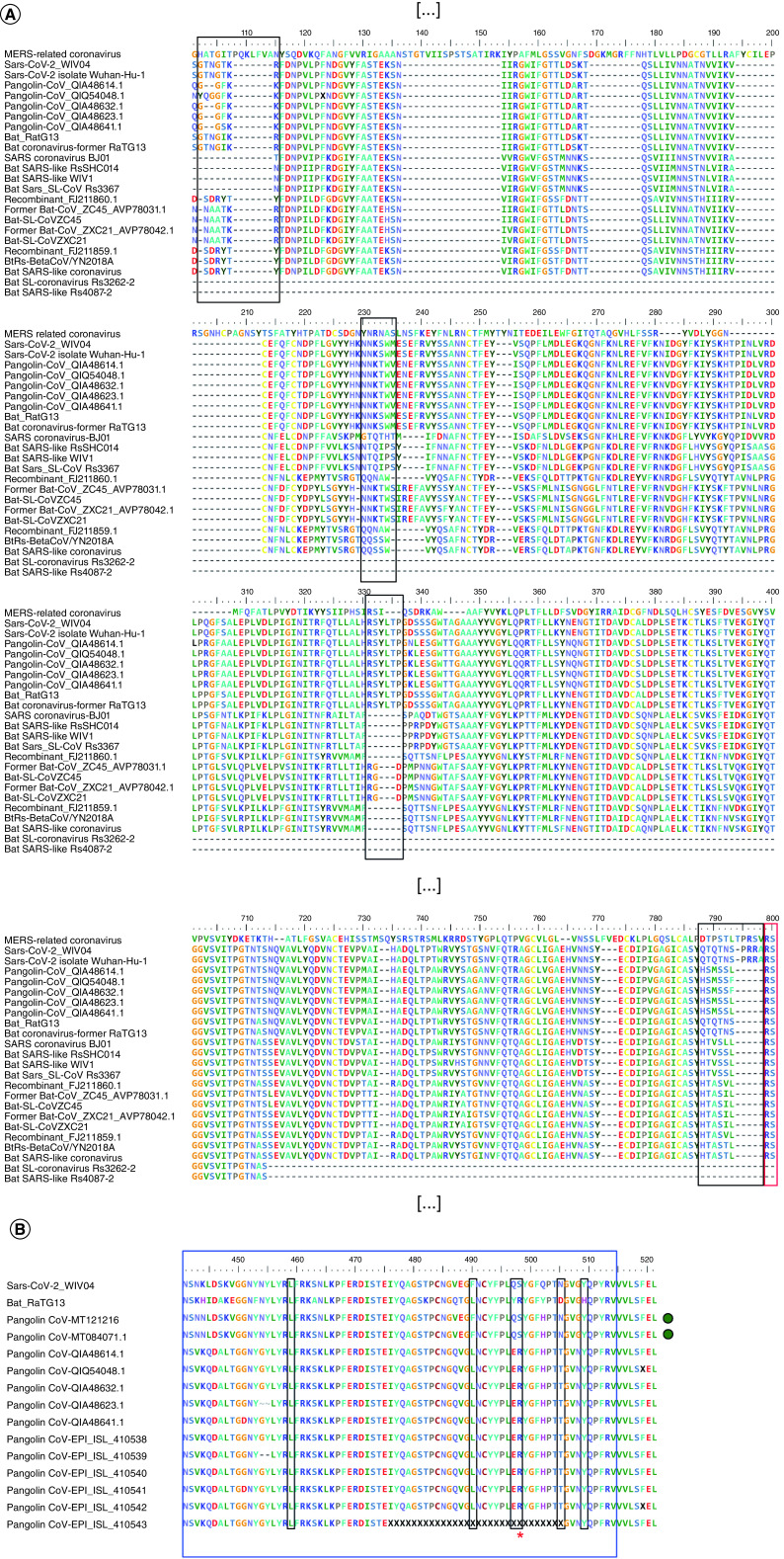
Polypeptide sequence alignment. **(A)** Alignment of the whole dataset of the S-proteins of SARS-CoV viruses. Respective names, accession numbers and additional information are reported in Table 1. Black rectangles: insertions -1 to -4, as reported by Zhou *et al.* [1] and Xiao *et al.* [5]. Red rectangle: S1/S2 cleavage site. Sequences were aligned using Muscle software [6]. Alignment accuracy was checked manually base by base using BioEdit v7.2.5 [7]. Part of the alignment not shown in this figure is indicated with: […]. **(B)** Alignment of the receptor-binding motif of the S-proteins of SARS-CoV-2, RaTG13 and of pangolin-CoVs. Blue rectangle: receptor-binding motif; black rectangles: positions of key amino acid residues involved in the interaction with human ACE2; red asterisk: additional key amino acid residue reported in Andersen *et al.* [8]; green circles: deposited sequences of pangolin-CoVs presenting a full asset of conserved key amino acid residues within the receptor-binding motif of SARS-CoV-2. If derived from the GISAID database (Table 1), S-protein sequences represented in this figure were predicted with orffinder (https://www.ncbi.nlm.nih.gov/orffinder/) using the deposited whole-genomes of their respective viruses as queries.

In Zhou *et al.* [1], polypeptide sequence alignment of the N-terminus of the S-proteins of SARS-CoV-2, RaTG13 and other bat coronaviruses demonstrated the existence of three main insertions (insertion-1, 72-GTNGTKR-78; insertion-2, 148-NNKSWM-153; insertion-3 246-RSYLTPGD-253) present only in SARS-CoV-2 and in RaTG13. These insertions are absent in most of the S-proteins of other coronaviruses, while they are partially similar for Bat-CoV-ZC45 (GenBank AVP78031.1), a recent SARS-like coronavirus collected from bats of the city of Zhoushan (China) during 2018 [3]. A fourth insertion (insertion-4, 677-QTNSPRRA-684) is present only in SARS-CoV-2 [4] and it is absent among the S-proteins of other SARS-coronaviruses, including Bat-CoV-ZC45 and RaTG13 (Figure 1A).

**Table 1.** Information related with the polypeptide sequences of the S-proteins analyzed in this research.

Viral strain name	Source	Accession	Submission date	DOI
MERS-related coronavirus	GenBank	ANF29184.1	30-04-2017	10.1093/infdis/jiw236
Sars-CoV-2_WIV04	GISAID	EPI_ISL_402124	11-01-2020	10.1038/s41586-020-2012-7
Sars-CoV-2 isolate Wuhan-Hu-1	GenBank	QHD43416.1	18-03-2020	10.1038/s41586-020-2012-7
Pangolin-CoV	ViPR/GenBank	QIA48614.1	23-04-2020	10.1016/j.cub.2020.03.022
Pangolin-CoV	ViPR/GenBank	QIQ54048.1	23-04-2020	10.1016/j.cub.2020.03.022
Pangolin-CoV	ViPR/GenBank	QIA48632.1	23-04-2020	10.1016/j.cub.2020.03.022
Pangolin-CoV	ViPR/GenBank	QIA48623.1	23-04-2020	10.1016/j.cub.2020.03.022
Pangolin-CoV	ViPR/GenBank	QIA48641.1	23-04-2020	10.1016/j.cub.2020.03.022
Pangolin-CoV^†^	GenBank	MT121216	18-05-2020	10.1371/journal.ppat.1008421
Pangolin-CoV^†^	GenBank	MT084071.1	23-04-2020	10.1002/bies.202000240
Pangolin-CoV	GISAID	EPI_ISL_410538	17-02-2020	10.1038/s41586-020-2169-0
Pangolin-CoV	GISAID	EPI_ISL_410539	17-02-2020	10.1038/s41586-020-2169-0
Pangolin-CoV	GISAID	EPI_ISL_410540	17-02-2020	10.1038/s41586-020-2169-0
Pangolin-CoV	GISAID	EPI_ISL_410541	17-02-2020	10.1038/s41586-020-2169-0
Pangolin-CoV	GISAID	EPI_ISL_410542	17-02-2020	10.1038/s41586-020-2169-0
Pangolin-CoV	GISAID	EPI_ISL_410543	17-02-2020	10.1038/s41586-020-2169-0
Bat_RatG13	GISAID	EPI_ISL_402131	24-03-2020	10.1038/s41586-020-2012-7
Bat coronavirus-former RaTG13	GenBank	QHR63300.1	24-01-2020	10.1038/s41586-020-2012-7
SARS coronavirus BJ01	GenBank	AAP30030.1	01-09-2009	10.1016/s1672-0229(03)01017-9
Bat SARS-like RsSHC014	GenBank	AGZ48806.1	22-11-2013	10.1038/nature12711
Bat SARS-like WIV1	GenBank	AGZ48828.1	22-11-2013	10.1038/nature12711
Bat Sars_SL-CoV Rs3367	GenBank	AGZ48818.1	22-11-2013	10.1038/nature12711
Recombinant FJ211860.1	GenBank	ACJ60703.1	31-12-2008	10.1073/pnas.0808116105
Former Bat-CoV_ZC45_AVP78031.1	GenBank	AVP78031.1	28-03-2018	10.1038/s41426-018-0155-5
Bat-SL-CoVZC45	GenBank	AVP78031.1	20-05-2020	10.1038/s41426-018-0155-5
Former Bat-Cov_ZXC21_AVP78042.1	GenBank	AVP78042.1	28-03-2018	10.1038/s41426-018-0155-5
Bat SL-CoVZXC21	GenBank	AVP78042.1	20-05-2020	10.1038/s41426-018-0155-5
Recombinant FJ211859.1	GenBank	ACJ60694.1	26-07-2016	10.1073/pnas.0808116105
BtRs-BetaCoV/YN2018A	GenBank	QDF43820.1	30-06-2019	10.3389/fmicb.2019.01900
Bat SARS-like coronavirus	GenBank	ATO98181.1	18-12-2017	10.1371/journal.ppat.1006698
Bat SL-coronavirus Rs3262-2	GenBank	AGZ48783.1	22-11-2013	10.1038/nature12711
Bat SARS-like Rs4087-2	GenBank	AGZ48802.1	22-11-2013	10.1038/nature12711

† Deposited sequences of pangolin-CoVs reporting a full asset of conserved key amino acid residues within the receptor-binding motif of the S-protein of SARS-CoV-2.

Supplementary data: Partial alignment among genomes of the coronaviruses RaTG13 and SARS-CoV-2 (accession numbers are available in Table 1) and the nucleotide sequence of BtCoV/4991 (Genbank KP876546.1) [9]. The polypeptide sequence is reported under the nucleotide sequences. Alignment of the nucleotide sequences is shown until the end of motif C [10] (red). Cyan, polymorphisms between BtCoV/4991 and SARS-CoV-2; bold: codon responsible of the amino acid substitution H-to-Y occurring between RaTG13 and SARS-CoV-2 (in cyan); green: interacting residues of the palm domain according with Gao *et al.* [10].

Among the first contributions investigating the existence of these four regions identified in the SARS-CoV-2 S-protein, a paper was submitted and subsequently withdrawn from BioRxiv (Pradan *et al.* 2020 – doi:10.1101/2020.01.30.927871). This contribution highlighted possible unconventional evolution of the virus and suggested total and partial identity of the four regions of the S-protein with motifs located on proteins from some strains of the human HIV-1 virus. In particular, V4, V5 and V1/V2 regions of the HIV-1 gp120 protein are similar, if not identical, to insertions -1 to -3 of the SARS-CoV-2 S-protein. Furthermore, another region of the HIV-1 gag protein shares part of identity to insertion-4 of the SARS-CoV-2 S-protein.

Soon after, another published investigation opposed these findings by demonstrating the distribution of these short polypeptide regions among proteomes of different organisms and of several viruses [5]. In the same paper, structural comparison of the SARS-CoV-2 S-protein and of the HIV-1 gp120 demonstrated lack of rationale for SARS-CoV-2 to obtain and mix structurally unrelated parts of HIV-1 proteins. For instance, while insertions -1 to -3 on the S-protein are structurally proximal and they may be involved in the same functional properties, on the HIV-1 gp120 V4 and V5 regions are separated by a LE loop and V1/V2, located on the opposite side of the protein, unlikely interacts with V4 and V5. Finally, insertion-4 on the S-protein is located too far from insertions -1 to -3 to be part of the same structural/functional unit, furthermore, the partially-identical region on the gag protein of HIV-1 is not involved in viral entry for this human virus. Absence of possible SARS-CoV-2/HIV-1 relationships is also evident from a polypeptide sequence alignment (Figure 1A): indeed, insertions -1 to -3 identified in the S-protein of SARS-CoV-2 are present among several wild coronaviruses infecting different animal hosts.

Based on these evidences, Xiao *et al.* [5] suggested the possibility of homologous recombination events in the course of infection of animal hosts to be at the base of the acquisition of the additional insertions within the mRNA sequence encoding the SARS-CoV-2 S-protein. Indeed, point mutations, deletions and insertions of both small and large regions of genomic sequences is renowned occurring among coronaviruses [11]. Recombination may explain a possible shift from bats to other animals. In support to this hypothesis, bats represent an animal reservoir for novel coronaviruses [12], and evolutionary analysis based on genomic sequences of coronaviruses suggested that a homologous recombination within the viral spike glycoprotein gene might have occurred between a bat coronavirus and a coronavirus with unknown origins involving, possibly, infection of an intermediate animal.

## In search of possible intermediate animal hosts

Analysis of the relative synonymous codon usage of SARS-CoV-2 and Bat-CoV-ZC45 demonstrated the existence of synonymous codons with two snake species that are widespread diffused in China: *Bungarus multicinctus* (many-banded krait) and *Naja atra* (Chinese cobra) [13]. This study advanced evidence on a homologous recombination within the viral spike glycoprotein gene of the SARS-CoV-2 and of a bat-CoV in the course of the infection in an intermediate animal. As reported in this study, SARS-CoV-2 may have more effectively used snake’s translation machinery than that of the other animals in the course of its transmission events to humans.

Another contribution suggested that other animal species might have represented a natural reservoir of SARS-CoV-2 coronavirus: CoVs isolates from dead Malayan pangolins (*Manis javanica* Desmarest) demonstrated up to 91.02% sequence identity with SARS-CoV-2 and RaTG13 at the whole genome level [14]. Phylogenetic analysis proposed that the S1-subunit of the S-protein of pangolin-CoVs is much more closely related to SARS-CoV-2 than to RaTG13. Indeed, before the SARS-CoV-2 outbreak, pangolins were the sole mammals other than bats that have been documented to host SARS-related coronaviruses [15].

Like other SARS-CoVs [16], including SARS-CoVs isolated from bats [17], the human SARS-CoV-2 uses angiotensin-converting enzyme 2 proteins (ACE2) as an entry receptor on mammalian cells; in particular, SARS-CoV-2 is capable to infect HeLa cells heterologously expressing ACE2 of human and of different mammals like civets, pigs and mice, demonstrating infection versatility of SARS-CoV-2 to different hosts [1]. Analysis of the receptor-binding motif, as part of the ACE2-receptor-binding domain, demonstrated conservation of every residue between SARS-CoV-2 and the pangolin-CoV, except for the unique Q500H substitution [14]. In particular, the key amino acid residues of the binding motif involved in ACE2-interaction, previously reported by Zhou *et al.* [1] (L455, F486, Q493, N501 and Y505), are conserved between SARS-CoV-2 and the pangolin-CoV. Contrary, in RaTG13 some of these residues are substituted: L486, Y493, D501 and H505 [1,14] (note: amino acid positions have been assigned based on Zhou *et al.* [1]). Furthermore, Zhang *et al.* [14] reported that part of the insertion-4 [5], which is unique for SARS-CoV-2 (-PRRARSV-) [1], is also absent in the pangolin virus, although insertion-1 is similar and insertions-2 and -3 are identical with SARS-CoV-2 and RaTG13 (Figure 1A). Taken together, these findings would have motivated possible generation of chimeric strains of coronavirus by recombination events happening within the receptor-binding domain of the S-protein of pangolins-CoVs or other RaTG13-like backbones.

In my analysis, polypeptide sequence alignment of the receptor-binding motif of the S-protein of SARS-CoV-2, RaTG13, and of the same S-proteins of pangolin-CoVs investigated by Zhang *et al.* [14] and deposited on 2020 on the ViPR database (https://www.viprbrc.org/brc/home.spg?decorator=corona; Table 1) demonstrates the presence of several substitutions (Figure 1B). In particular, except for L455 and Y505 (L459 and Y509 in the figure), three out of the five key amino acid residues of the motif involved in ACE2-binding [1,14] are not conserved between CoVs of human and pangolin. However, a parallel investigation from a comparative analysis of genomic data generated from the database of the same pangolin sequencing projects used by Zhang *et al.* [14] (SRR10168377 and SRR10168378 - NCBI BioProject PRJNA573298 [15]), identified conservation of the five key amino acid residues and included a sixth conserved residue (S494) [8]. In addition, gap filling of the whole genome sequence of the pangolin-CoV-2020 project, made of two pangolin-CoV samples from the same dataset [15] and of one additional sample from a newly collected dead pangolin, returned an updated result (GenBank MT121216), which S-protein conserved all six key amino acid residues of the receptor-binding motif [18].

Even though it is undisputable that deeper [8] and updated investigations [18] would provide results that are more reliable, the accuracy of assembling data from pangolin-CoVs has been widely questioned [19–21], claiming further analysis to prove the correctness of the assembled data from pangolin-CoVs. For instance, findings from Zhang *et al.* [14], Andersen *et al.* [8] and Liu *et al.* [18] rely on the same dataset generated the year before by Liu *et al.* [15], which started from a singular batch of dead pangolins [22]. In addition, genome sequencing from CoVs sampled from different pangolins (GISAID EPI_ISL_410538 - EPI_ISL_410543) [23] predicted S-proteins, which key amino acids of the receptor-binding motif are not conserved with the binding-motif of the S-protein of SARS-CoV-2 (Figure 1B).

## The origins of RaTG13

Since its release in *Nature* on 3 February 2020, the research of Zhou *et al.* [1] was object of a long list of critical comments, among which, several were wondering about the origins of RaTG13. In fact, independently from the multiple investigations reporting evidences of the recombinatorial origins of SARS-CoV-2 from CoVs infecting different animals [5,8,11–18,23], the genome of the bat-CoV RaTG13 was revealed to be the closest phylogenetic relative for the human coronavirus [1,24], but a reliable traceability of the origins of its sample was missing from Zhou *et al.* [1].

Among the several databases where RaTG13 has been deposited [NGDC (https://bigd.big.ac.cn/) accession number: GWHABKP00000000, released 30-01-2020; GISAID (gisaid.org) accession number: EPI_ISL_402131, submitted 24-01-2020, collected on 24-07-2013 (date more recently updated since 24 March 2013); GenBank (https://www.ncbi.nlm.nih.gov/) accession number: MN996532.1, submitted from 29 January 2020 to 24 March 2020 after several revisions] no evidence is documented about the submission of this viral genome earlier than 2020. The only evidence relates from a partial sequence of a bat coronavirus reported in 2016 and isolated in 2013 from bat feces sampled in an abandoned mine of Mojiang (Yunnan county, China), named BtCoV/4991 (Genbank KP876546.1) [9] that shows 100% identity with a codifying ORF for the RNA-dependent RNA-polymerase (RdRp) of RaTG13 [19,25]. However, no references to BtCoV/4991 from Ge *et al.* [9] are reported in Zhou *et al.* [1], but rather an indication that RaTG13 was ‘a short region of RdRp from a bat coronavirus … which was previously detected in *Rhinolophus affinis’*. Although I am aware about the fact that sequencings that are more recent are deposited right upon submission, and naming may be updated accordingly, which may have been more likely the case for RaTG13 from Zhou *et al.* [1], facing the seriousness of the COVID-19 pandemic, referencing to findings from Ge *et al.* [9] of BtCoV/4991 may have deserved an undisputable importance. Indeed, an investigation published in concomitance with Zhou *et al.* [1] already indicated the coronavirus strain named BtCoV/4991 to be at the origin of an independent introduction of the SARS-CoV-2 virus from bat to human [26].

Comparing SARS-CoV-2 (GISAID EPI_ISL_402124), BtCoV/4991 shows a partial identity of 99.99%, which is not total because of the existence of five polymorphisms (Supplementary data). Although three out of these five polymorphisms do not alter the amino acid composition of their related polypeptide sequence, 2 of them (C15595T and C15597T, position assigned from the nucleotide sequence of SARS-CoV-2) cause a histidine-to-tyrosine substitution 33 residues upstream of the RdRp-motif C, in a region of the palm domain surrounded by RNA-interactive residues [10]. Whether the specific positioning of this amino acid substitution may influence or not RdRp activity is not documented. However, the identification of this substitution within the palm domain, which forms part of the nucleic acid binding tunnel [10], may address studies to compare binding capacities between RdRps of RaTG13 and SARS-CoV-2.

Molecular evidences from the identity of the BtCoV/4991 isolate with RaTG13 and SARS-CoV-2 add to the documented literature that the earliest cases of the COVID-19 disease may not have been connected with the local seafood market of Wuhan city [1]. Indeed, it was rather suggested that SARS-CoV-2 coronavirus came into the marketplace where the first cases described by Zhou *et al.* [1] have been reported, before it came out of that [27].

Up to now the origins of SARS-CoV-2 are still unknown. In the last months, the WHO launched the Scientific Advisory Group for the Origins of Novel Pathogens (SAGO) [28], where 26 selected scientists are involved to shed more light on the origins of the COVID-19 pandemic.

## Reported evidences of possible alternative origins of SARS-CoV-2

Analyzing the polypeptide sequence of the S-protein, the SARS-CoV-2 insertion-4, that is renown as a possible furin recognition site (-PRRARSV-), is absent in any animal CoV, including RaTG13, but it is present, although not identical (-PRSVRSV-), only in the MERS coronavirus (GenBank ANF29184.1, Table 1) (Figure 1A). This short motif may represent a specific feature for CoV-strains that are renowned infecting a human host [4], where the -PRRARSV- motif, in specific, seems to be up to now unique for the sole SARS-CoV-2 coronavirus.

Analyzing the respective nucleotide sequence, Segreto and Daigin [19] noticed that the 5´-TCCTCGGCGGGC-3´ insertion coding for the furin recognition site of SARS-CoV-2 is not in frame with the rest of the sequence when compared with the S-protein of the CoV of pangolin and RaTG13. In addition, this insertion includes a FauI restriction site. By their observation, the authors excluded the possibility that such insertion could have been the result of a polymerase slippage or by releasing and re-priming, as these rearrangements are expected to maintain the reading frame. Accordingly, evidences reported in the *Journal of Medical Viology* [29] specified that although millions of SARS-CoV-2 cases have been globally recorded (as mentioned in the introduction), clinical isolates of SARS-CoV-2 have not indicated any further recombination in the proximity of the furin recognition motif of the S-protein, which is unconventional with the general recombination model expected for other coronaviruses. In their paper, Segreto and Deigin [19] suspected that the additional nucleotides responsible for the existence of the -PRRARSV- motif for SARS-CoV-2 may have been the result of an artificially insertion to facilitate manipulation of the SARS-CoV-2 coronavirus to prepare pan-CoV vaccines therapies [30–33], or to improve capabilities of the virus to infect cells of different animal origins for research purposes [34]. Interestingly, in their analysis Segreto and Daigin [19] compared the incomplete pangolin-CoV sequence (GenBank MT084071.1) from the isolate MP789, which was updated in a further genomic assembling to GenBank MT121216 [18]. The nucleotide composition of the CDS for this S-protein is partially different from other sequences deposited for pangolin-CoVs [14,23]; however, the evidence discussed by Segreto and Daigin [19] of a not-in-frame nucleotide insertion is coherent for any deposited CDS coding S-proteins from different pangolin coronaviruses.

Despite motivations from Segreto and Daigin [19] are convincing, mutations, in general, as well as recombination events within genes coding S-proteins of coronaviruses are well documented [11]. Recently, the divergence of Omicron among the most recent strains of SARS-CoV-2 coronavirus [35], demonstrated an extreme plasticity of the S-protein (PDB 6VSB), where among the several amino acid substitutions, some affect the structure of the furin recognition motif [36]. In addition, a past investigation on insect membrane proteins demonstrated occurrence of mRNA-editing insertion that are not-in-frame of up to 15 nucleotides within transcripts coding insect transmembrane proteins like TRP-channels [37]. Although this research did not target viral transcripts, it brought up evidences that general mechanisms of mRNA-editing occur naturally among multiple organisms [38] and, interestingly, they appear even more extensive in invertebrates [37,39]. Although some studies report insects that may transfer coronaviruses [40] up to now there are no evidences reporting insects as vectors of coronaviruses where the pathogen may replicate to be transmitted to a human host [41]. However, the occurrence in insects of RNA-editing mechanisms adding nucleotides not-in-frame to coding transcripts and the existence for the S-protein of the SARS-CoV-2 coronavirus of transcripts re-conducting to similar mechanisms are compelling evidences. In this scenario, a wider reservoir of animal hosts for SARS-CoV-2 coronavirus deserves to be investigated among vertebrates and, possibly, invertebrates, to validate the existence of a natural adaptation and transmission mechanisms converting an ancestral SARS-CoV from an animal to a human pathogen.

## Conclusion

For a better understanding of the evolutionary aspects of SARS-CoV-2 to the improvement of its containment strategies, and to avoid rise of disruptive theories from the public domain, more evidences are needed to shed light on the introduction of the virus from bat to human. To this target, I think that additional sequencing projects on pangolin CoVs will add knowledge of any possible relationship of the pangolin host with SARS-CoV-2, to understand weather pangolin is the main or only one among the possible animal intermediates in the transmission of the virus to humans. The evidence of the existence in the S-protein of an unusual furin recognition motif, which nucleotide coding sequence seems to be far from being originated by a natural recombination [19,29] leaded to the hypothesis of SARS-CoV-2 origins related with a possible artificial manipulation of an ancestral coronavirus. However, based on the previous studies [37], RNA-editing mechanisms adding several nucleotides not-in-frame can occur naturally. Although speculative, we cannot exclude that in phase of infection of animals from the coronavirus reservoir, such editing events may have interfered with the RNA of the virus. Investigations are needed to validate if these mechanisms may occur in possible SARS-CoV hosts, like, for instance, the bat *R. affinis*, the pangolin *M. javanica*, other vertebrates [13] or, although possibly, invertebrates somehow involved in the transmission of the virus [40]. This would help to add evidences of the virus adaptation from animals to human.

## Future perspective

Evidences suggest versatility of SARS-CoV-2 adapting among various possible hosts and, within its hosts, the potentials for this virus to diverge to novel strains that may be more (or less) detrimental after their hosts’ infection. Actually, social distancing, development of updated vaccines and exploring possible pharmacological treatments represent the sole measures to cope with this phenomenon and to reduce the diffusion of SARS-CoV-2. However, the efficacy of these measures is still limited. Since the current large-scale diffusion of the virus and the facility of its transmission, co-existence with the virus represents the sole option that I foresee for the next 5–10 years from now. Initiatives shedding more light on the origins of SARS-CoV-2 would make more predictable the possible of adaptation of this virus to new species, to help preventing the divergence of novel strains that may be even more dangerous for human health.

**Executive summary**

**Investigating the origins of the amino acid insertions within the SARS-CoV-2 S-protein**

- The identification of four short polypeptide insertions into the amino acid sequence of the S-protein of SARS-CoV-2 sparked a debate in the scientific community when controversial findings proposed their possible relations with proteins from HIV-1.
- The most accredited hypothesis suggest that homologous recombination within the viral spike glycoprotein (S-protein) gene might have occurred between a coronavirus from bats (RaTG13) and a coronavirus with unknown origins involving, possibly, infection of an intermediate animal host.

**In search of possible intermediate animal hosts**

- Apart from bats, other animals may have been a reservoir of hosts for the SARS-CoV-2 coronavirus, including snakes and the Malayan pangolin *Manis javanica*.
- For the pangolin, published evidences reported identities in the receptor-binding motifs of the S-proteins from several CoVs and the S-protein of SARS-CoV-2. In addition, these reports indicate the existence of identities within the furin recognition motif present in the S-protein insertion-4.
- Performing a polypeptide sequence alignment starting from the same sequences, such identities cannot be confirmed. All sequencing investigations up to now conducted on pangolin coronaviruses started from viral samples collected from the same pangolin individuals but deposited different assembling of the S-protein.

**The origins of RaTG13**

- Since the outbreak of SARS-CoV-2, the bat CoV RaTG13 has been reported as its most identical coronavirus (Zhou *et al.* [1]). However, in this report, accurate referencing is missing for samplings and sequencing deposition of the first isolate named BtCoV/4991, which is 99.99% identical to RaTG13 and SARS-CoV-2 and was identified on 2013.
- The existence of BtCoV/4991 add to the more dated documented origins of SARS-CoV-2, that anticipates its outbreak of late 2019 when this strain of coronavirus was isolated from seafood street-sellers of Wuhan city (China).

**Reported evidences of possible alternative origins of SARS-CoV-2**

- The existence of the furin recognition motif making the virus suitable for a human host, and the identification of a cloning restriction site within its nucleotide sequence, motivated hypothesis of possible artificial origins of SARS-CoV-2 coronavirus.
- The hypothesis of artificial origins was further supported by proven absence of recombinatorial events in the proximity of this motif in the course of the large-scale infection of the virus. However, for coronaviruses in general, mutations and recombinations are frequent.
- Although reported from invertebrates, published evidences demonstrated the existence of natural mechanisms of RNA-editing adding up to 15 nucleotides not-in-frame within coding sequences of transmembrane proteins, that may justify occurring of similar events for the SARS-CoV-2 coronavirus.
- Evidences of adaptation of SARS-CoV-2 to humans from strains of coronavirus with a reservoir of hosts among various animals and the plasticity of the S-protein of these viruses suggest more complicated origins for the pathogen.
- Future investigation of other possible host candidates for SARS-CoV-2 will help understanding its adaptability to different animals to help preventing worsening of the ongoing pandemic situation.
