# Supplementary material for: Reviewing findings on the polypeptide sequence of the SARS-CoV-2 S-protein to discuss the origins of the virus
Source: Future Virol. 2022 Apr 5;0(0):10.2217/fvl-2021-0233. doi: 10.2217/fvl-2021-0233 (PMC8982992; doi:10.2217/fvl-2021-0233)
Supplement: Supplementary file 1 [file supplementary_figure.pdf]

```
             ....|....| ....|....| ....|....| ....|....| ....|....| ....|....|
                 15305      15315      15325      15335      15345      15355
RaTG13       AAATGTGATA GAGCCATGCC TAACATGCTT AGAATTATGG CCTCACTTGT TCTTGCTCGC
SarsCoV2     AAATGTGATA GAGCCATGCC TAACATGCTT AGAATTATGG CCTCACTTGT TCTTGCTCGC
BtCOV4991    ---------- ---------- ---------- ---------G CCTCACTTGT TCTTGCTCGC
                                                          A  S  L   V  L  A  R

             ....|....| ....|....| ....|....| ....|....| ....|....| ....|....|
                 15365      15375      15385      15395      15405      15415
RaTG13       AAACATACAA CGTGCTGTAG CTTGTCACAC CGTTTCTATA GATTAGCTAA TGAGTGTGCT
SarsCoV2     AAACATACAA CGTGTTGTAG CTTGTCACAC CGTTTCTATA GATTAGCTAA TGAGTGTGCT
BtCOV4991    AAACATACAA CGTGCTGTAG CTTGTCACAC CGTTTCTATA GATTAGCTAA TGAGTGTGCT
               K  H  T   T  C  C   S  L  S  H   R  F  Y   R  L  A   N  E  C  A

             ....|....| ....|....| ....|....| ....|....| ....|....| ....|....|
                 15425      15435      15445      15455      15465      15475
RaTG13       CAAGTATTGA GTGAAATGGT CATGTGTGGC GGTTCACTAT ATGTTAAACC AGGTGGAACC
SarsCoV2     CAAGTATTGA GTGAAATGGT CATGTGTGGC GGTTCACTAT ATGTTAAACC AGGTGGAACC
BtCOV4991    CAAGTATTGA GTGAAATGGT CATGTGTGGC GGTTCACTAT ATGTTAAACC AGGTGGAACC
               Q  V  L   S  E  M   V  M  C  G   G  S  L   Y  V  K   P  G  G  T

             ....|....| ....|....| ....|....| ....|....| ....|....| ....|....|
                 15485      15495      15505      15515      15525      15535
RaTG13       TCATCAGGAG ATGCCACAAC TGCTTATGCT AATAGTGTCT TTAACATTTG TCAAGCTGTT
SarsCoV2     TCATCAGGAG ATGCCACAAC TGCTTATGCT AATAGTGTTT TTAACATTTG TCAAGCTGTC
BtCOV4991    TCATCAGGAG ATGCCACAAC TGCTTATGCT AATAGTGTCT TTAACATTTG TCAAGCTGTT
               S  S  G   D  A  T   T  A  Y  A   N  S  V   F  N  I   C  Q  A  V

             ....|....| ....|....| ....|....| ....|....| ....|....| ....|....|
                 15545      15555      15565      15575      15585      15595
RaTG13       ACGGCCAATG TTAATGCACT TTTATCTACT GATGGTAACA AAATTGCCGA TAAGCACGTC
SarsCoV2     ACGGCCAATG TTAATGCACT TTTATCTACT GATGGTAACA AAATTGCCGA TAAGTATGTC
BtCOV4991    ACGGCCAATG TTAATGCACT TTTATCTACT GATGGTAACA AAATTGCCGA TAAGCACGTC
               T  A  N   V  N  A   L  L  S  T   D  G  N   K  I  A   D  K H/Y  V
```

```
               ....|....| ....|....| ....|....| ....|....| ....|....| ....|....|
                   15605      15615      15625      15635      15645      15655
RaTG13         CGCAATTTAC AACACAGACT TTATGAGTGT CTCTATAGAA ATAGAGATGT TGACACAGAC
SarsCoV2       CGCAATTTAC AACACAGACT TTATGAGTGT CTCTATAGAA ATAGAGATGT TGACACAGAC
BtCOV4991      CGCAATTTAC AACACAGACT TTATGAGTGT CTCTATAGAA ATAGAGATGT TGACACAGAC
                 R  N  L   Q  H  R   L  Y  E  C   L  Y  R   N  R  D   V  D  T  D

               ....|....| ....|....| ....|....| ....|....| ....|....| ....|....|
                   15665      15675      15685      15695      15705      15715
RaTG13         TTTGTGAATG AGTTTTACGC ATATTTGCGT AAACATTTCT CAATGATGAT ACTTTCTGAT
SarsCoV2       TTTGTGAATG AGTTTTACGC ATATTTGCGT AAACATTTCT CAATGATGAT ACTCTCTGAC
BtCOV4991      TTTGTGAATG AGTTTTACGC ATATTTGCGT AAACATTTC- ---------- ----------
                 F  V  N   E  F  Y   A  Y  L  R   K  H  F   S  M  M   I  L  S  D

               ....|....| ....|....| ....|....| ....|....| ....|....| ....|....|
                   15725      15735      15745      15755      15765      15775
RaTG13         GATGCTGTTG TGTGTTTCAA TAGCACTTAT GCATCTCAAG GTCTAGTGGC TAGCATAAAG
SarsCoV2       GATGCTGTTG TGTGTTTCAA TAGCACTTAT GCATCTCAAG GTCTAGTGGC TAGCATAAAG
BtCOV4991      ---------- ---------- ---------- ---------- ---------- ----------
                 D  A   V  V  C   F  N
```
